# When a Plant Resistance Inducer Leaves the Lab for the Field: Integrating ASM into Routine Apple Protection Practices

**DOI:** 10.3389/fpls.2017.01938

**Published:** 2017-12-04

**Authors:** Brice Marolleau, Matthieu Gaucher, Christelle Heintz, Alexandre Degrave, Romain Warneys, Gilles Orain, Arnaud Lemarquand, Marie-Noëlle Brisset

**Affiliations:** ^1^IRHS, INRA, Agrocampus-Ouest, Université d’Angers, SFR 4207 QUASAV, Beaucouzé, France; ^2^Unité Expérimentale Horticole, INRA, Beaucouzé, France

**Keywords:** plant resistance inducer, acibenzolar-*S*-methyl, apple scab, pest management, cultivar, fire blight, systemic acquired resistance

## Abstract

Plant resistance inducers, also called elicitors, could be useful to reduce the use of pesticides. However, their performance in controlling diseases in the field remains unsatisfactory due to lack of specific knowledge of how they can integrate crop protection practices. In this work, we focused on apple crop and acibenzolar-*S*-methyl (ASM), a well-known SAR (systemic acquired resistance) inducer of numerous plant species. We provide a protocol for orchard-effective control of apple scab due to the ascomycete fungus *Venturia inaequalis*, by applying ASM in combination with a light integrated pest management program. Besides we pave the way for future optimization levers by demonstrating in controlled conditions (i) the high influence of apple genotypes, (ii) the ability of ASM to prime defenses in newly formed leaves, (iii) the positive effect of repeated elicitor applications, (iv) the additive effect of a thinning fruit agent.

## Introduction

Numerous pathogens and pests attack apple trees (*Malus* × *domestica*), causing important losses in yield and quality harvest. They include, among others, *Venturia inaequalis* (apple scab), *Dysaphis plantaginea* (apple rosy aphid), *Cydia pomonella* (codling moth), *Erwinia amylovora* (fire blight), *Podosphaera leucotricha* (powdery mildew). Apple is one of the highest pesticide consuming crops (more than 30 treatments per year in France in conventional apple production systems) with a majority of treatments dedicated to the control of apple scab.

The intensive use of pesticides raises many questions concerning selection of pesticide-resistant strains, environmental impact and human health risks (Alavanja et al., 2004; Ma and Michailides, 2005; Chambers et al., 2014). Among strategies to reduce pesticides, the use of plant resistance inducers (PRIs, also called elicitors or plant defense activators) appears as a potential option to face the phytosanitary issues of conventional agricultural practices (Thakur and Sohal, 2013; Walters et al., 2013). These agents include a variety of chemical (Bektas and Eulgem, 2015) or biological (Wiesel et al., 2014) stimulators able to activate plant defenses by exogenous application. Depending on their very nature, they either act as non-self determinants (mimicking MAMP – Microbe Associated Molecular Patterns – or DAMP – Damage Associated Molecular Patterns – general elicitors) perceived by pattern recognition receptors (PRR) present at the cell surface (Zipfel, 2008; Sanabria et al., 2010; Henry et al., 2012) or mimic plant downstream signaling molecules such as phytohormone analogs or derivates (Pieterse et al., 2012; Derksen et al., 2013). Exogenous application of PRIs aims at leading the plant defense system into an induced or primed state. The latest is considered as a poised state of defenses resulting in a stronger/faster induction of defense responses upon subsequent biotic or abiotic stress (Conrath, 2011). The resulting plant defense responses include cell wall fortification as well as production of antimicrobial compounds such as PR (Pathogenesis Related)-proteins, phytoalexins or ROS (Reactive Oxygen Species) (Hammond-Kosack and Jones, 1996).

The induced resistance generated by PRIs is intended to be broad spectrum and long-term efficient, but the resulting protection effect is not always as strong as expected, even in controlled conditions. Their success is most probably influenced by a great number of factors (genotype, environment, crop nutrition, prior induced state in field) and important studies remain to be done to optimize their use and their efficiency, especially when applied in field (Walters et al., 2005, 2013). However, PRIs are seen as a hopeful strategy in light of the current awareness of the need to reduce the use of pesticides.

One of the most studied PRIs is acibenzolar-*S*-methyl (ASM), a salicylic acid functional analog belonging to the benzothiadiazole (BTH) family. It is a synthetic SAR (Systemic Acquired Resistance, for a review see Gozzo and Faoro, 2013) inducer for crop protection, registered under the names Bion^®^ or Actigard^®^ (Syngenta). ASM efficiency has been reported in many crop species for its performance in controlling a large number of pathogens and/or in inducing or priming multiple immune responses (Gozzo and Faoro, 2013; Thakur and Sohal, 2013; Walters et al., 2013; Bektas and Eulgem, 2015). Regarding the apple case, several works demonstrated its ability to protect against apple scab (Bengtsson et al., 2006, 2009), fire blight (Brisset et al., 2000; Maxson-Stein et al., 2002; Baysal and Zeller, 2004; Dugé de Bernonville et al., 2014; Aćimović et al., 2015), *Alternaria* leaf blotch (Sofi et al., 2012), post-harvest diseases (Quaglia et al., 2011), and some demonstrated a strong correlation between performance in controlling diseases and defense induction. Additional studies are however requested to know how to integrate ASM in real disease management program in the orchard.

In the present study, we evaluated the performance of ASM against natural apple scab infection in orchard, when integrated in a classical but soft fungicide management program. In an attempt to fully exploit the potential of ASM, we investigated in controlled conditions several factors that may affect the expression of induced resistance after its application on apple, i.e., responsiveness of host genotype, persistence of action and effect of cumulative treatments. We also analyzed the combined effect of ASM with some other agricultural inputs commonly used in orchard to reveal their potential synergistic or antagonistic effect on induced resistance. Inductions of defense genes as well as control of apple scab and fire blight after artificial inoculation were assessed in order to explore various features of the product.

## Materials and Methods

### Biological Material and General Procedures

#### Plant Material

The field trial was conducted on 7 year-old ‘Golden Reinders’ apple trees, *Malus × domestica* Borkh, grafted on ‘M9 EMLA’ rootstocks and organized in 3 blocks of 4 plots of 5 rows of 13 trees (**Supplementary Figure S1**). Blocks were separated from one another by hedgerows to promote the presence of auxiliary fauna and to avoid cross-contamination with fungicides and PRIs.

Greenhouse experiments were performed on apple seedlings (4–6 leaves) from open-pollinated cv. Golden Delicious and on scions of five *Malus × domestica* genotypes chosen for their contrasted susceptibility to apple scab: Elstar (moderately susceptible), Golden Delicious (susceptible), Granny Smith (susceptible), Fuji (susceptible), Pink Lady (very susceptible) (Washington et al., 1998). Plants were grown under greenhouse conditions (natural photoperiod supplemented with artificial light if needed, 17°C night and 23–25°C day according to the sun light).

### Compounds and Sprayers

The compounds used in this study are listed in **Table 1**. In the field protection assay, compounds were solubilized in tap water and sprayed with an orchard sprayer S21 (Pulverization S21, Samazan, France) equipped with ATR80 nozzles (Albuz, Evreux, France). For greenhouse experiments, compounds were solubilized in reverse osmosis water and sprayed to runoff on entire plants with a spray gun Aeryo-1.4 (Deltalyo, Mably, France).

**Table 1:** Characteristics of compounds.

Commercial name	Firm	Active ingredient (concentration)	Code	General use	Concentration used^∗^
Bion^®^ 50 WG	Syngenta	Acibenzolar-S-methyl (50% w/w)	ASM	PRI	0.4 g/L
MaxCel^®^	Valent Biosciences	6-benzyladenine (1.9% w/v)	6BA	Thinning agent	15 mL/L
Pitpom^®^	Yaravita France	Calcium (20% w/w)	Ca	Foliar fertilizer	20 mL/L
Rhodofix^®^	Nufarm	Naphthalene acetic acid (1% w/w)	NAA	Thinning agent	1.5 g/L

*^∗^Concentration of commercial product.*

### Plant Protection Assays

#### Field Assay

The one-year experiment consisted in the comparison of three pest management strategies (**Table 2**) during the apple scab primary infection period (April–May) under natural contamination: an Integrated Pest Management (IPM) using conventional pesticide applications whatever the apple scab risk, a light IPM in which curative pesticides were only applied in case of proven severe risks, and a light IPM + ASM following the same decision rules as for light IPM but complemented with ASM applications every 6-8 days during the period of primary contamination. The fungicide schedule was performed according to two complementary decision tools: The Mills model (Mills and LaPlante, 1951) which predicts light, moderate, or severe risks according to weather forecasts (temperature and duration of leaf wetness) was used for preventive fungicides treatments; and the Pulsowin 3.1 software (Pulsonic, Orsay, France), which identifies proven risks thanks to local weather records (hourly climatic data), was used for curative fungicides treatments. The experimental treatment (light IPM + ASM) and the two control treatments (IPM and light IPM) were randomly applied on 3 plots of each of the 3 blocks of the orchard (**Supplementary Figure S1**). Symptoms were assessed on leaves and fruits the 30th of June. Respectively 4 shoots and 20 fruits were examined per trees on 11 trees of the central row of each plot. The first and the last tree of each central row were excluded to avoid edge effects. At harvest (24th of September), all fruits were collected on two trees of the central rows of each plot. Disease incidence was calculated as the percentage of the number of infected shoots or fruits/total number of observed shoots or fruits per tree (30th of June) or per plot (24th of September). Disease severity was calculated as the percentage of infected leaves per infected shoot and per tree. Fruits underwent an automatic grading (MSM2000, Greefa, Geldermasen, Holland) before their weighing, caliber per caliber.

**Table 2:** Diseases management strategies against apple scab.

	Apple scab prediction risks		
	Light	Moderate	Severe
IPM	X	X	X
Light IPM			X
Light IPM + ASM	ASM every 6–8 days		X

*X, preventive and/or curative fungicide treatments; ASM, acibenzolar-*S*-methyl; IPM, Integrated Pest Management.*

#### Greenhouse Assays

In both fire blight and scab protection tests, the experimental design was a randomized block of 3 plots of 10 seedlings per treatment. Inoculations were performed 3 and/or 10 days post treatment (dpt) and each assay was repeated in two independent biological experiments at least (details are given in figure legends).

##### Fire blight

Inoculation was performed as previously described (Dugé de Bernonville et al., 2014). Briefly, the virulent CFBP1430 strain of *E. amylovora* (Paulin and Samson, 1973) was cultivated at 26°C overnight on solid King’s B medium (King et al., 1954). Bacterial suspensions were prepared in reverse osmosis water at 10^8^ colony-forming units (CFU)/mL. The morning of inoculation, the youngest leaf of each plant was wounded by a double incision perpendicular to the midrib near the petiole, and the bacterial suspension was sprayed 4 h later on entire seedlings to runoff with a pressurized hand sprayer. Symptoms, i.e., evolutive necrosis starting from the inoculation site, were assessed 2 and 3 weeks later. Disease incidence was expressed as the percentage of the number of infected plants/total number of inoculated plants per plot and relative to the mean of the three plots of water control.

##### Scab

The isolate 104 of *V. inaequalis* (Guillaumès et al., 1995) was chosen for apple scab protection tests. Conidial suspensions were prepared from diseased leaves of apple seedlings in reverse osmosis water, calibrated to 1.5 × 10^5^ conidia/mL and sprayed on entire seedlings to runoff with a pressurized hand sprayer. After inoculation, plants were maintained 2 days in high-humidity chambers (darkness, 18°C, 90–100% relative humidity) and incubated afterward in semi-controlled conditions with a photoperiod (16 h light/8 h dark, constant temperature of 18°C, 70% of relative humidity). Symptoms, i.e., sporulating lesions, were assessed 2 and 3 weeks later. Disease incidence was calculated as the percentage of the number of infected plants/total number of inoculated plants per plot and relative to the mean of the three plots of water control.

### Defense Induction

The experimental set-up was a randomized block of *n* plots of five plants per treatment (one plot per treatment and per sampling date after treatment) and an additional plot of five plants remaining untreated (day 0 sampling date). Each block was sprayed with compounds or mock. In each block, some plots received an additional hydrogen peroxide (Hp, 40 mM) to runoff with a trigger sprayer 24 or 48 h before sampling in order to mimic a pathogen attack as previously described (Dugé de Bernonville et al., 2014). At each sampling date (day 0 and day 3 or 10 depending on experiments), five youngest expanded leaves per plot (treated with Hp or not) were collected, pooled, frozen in liquid nitrogen, and kept at -80°C until extraction. Each experiment was repeated twice.

RNA extraction, reverse-transcription, and quantitative real-time PCR were performed as previously described (Vergne et al., 2014) using the same patented set of primers for the 28 defense genes and 3 reference genes (Brisset and Dugé de Bernonville, 2011) or a selection of them (*PR2, PR4* and *CSL* for defense genes and *GAPDH* and *TuA* for reference genes). Relative changes in defense gene expression (log_2_ ratio) were calculated using the 2^-ΔΔC_T_^ method (Schmittgen and Livak, 2008) and the 3 or 2 internal reference genes for normalization (Vandesompele et al., 2002) and relative to the mean values obtained from untreated plants sampled at the beginning of each experiment or water control plants sampled the same day (as indicated in figure legends).

### Data Analyses

For plant protection assays and fruit yield, statistical analyses were performed with a nonparametric rank-based statistical test (Kruskal–Wallis) with Fisher’s Least Significant Difference (LSD) test for pairwise comparisons. χ^2^ analysis was used for comparison of distributions of fruit calibers. For gene expression, a Principal Component Analysis (PCA) was applied for genotype comparison, using an untreated sample of Elstar as a unique calibrator for the calculation of the log_2_ ratio of each defense gene. One-way analyses of variance (ANOVA) were conducted to compare treatments followed by LSD pairwise comparison tests, using log_2_ expression data relative to the mean of untreated tissues at day 0 for each defense gene. For each analysis, significance was reported at α = 0.05 significance level.

## Results

### Integration into Orchard Management System

**FIGURE 1:**
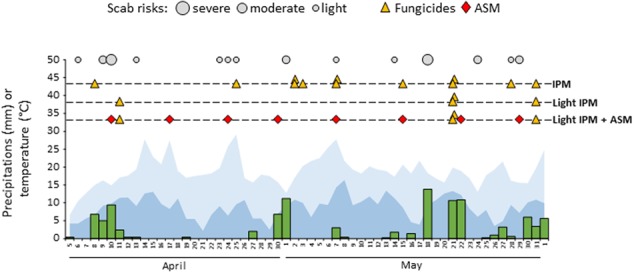
Weather data, model output, fungicides, and PRI schedule in the three pest management strategies: IPM, light IPM and light IPM + ASM during the season of primary apple scab contamination. Rain, minimal temperature, and maximal temperature are represented in green, dark blue and light blue respectively. Scab risks correspond to proven risks of apple scab and are represented according to their amplitude (light, moderate and severe). Fungicide treatments are represented by yellow triangles, ASM applications by red diamonds. ASM, acibenzolar-*S-*methyl; IPM, integrated pest management.

Weekly scheduled applications of ASM superimposed to a light application of fungicides (light IPM + ASM) during the natural apple scab primary infection period was compared to a classical IPM (positive control) and to the light IPM (negative control). During the period, 14 proven risks were recorded in the experimental orchard: eight light, four moderate, and two severe risks (**Figure 1**). These risks were covered by 12 preventive or curative treatments in IPM, whereas only the two severe and the last moderate risk of the period were covered by four curative treatments in light IPM (±ASM). The schedule of all commercial agricultural inputs including pesticides used in the three management strategies is listed in **Supplementary Table S1**.

**FIGURE 2:**
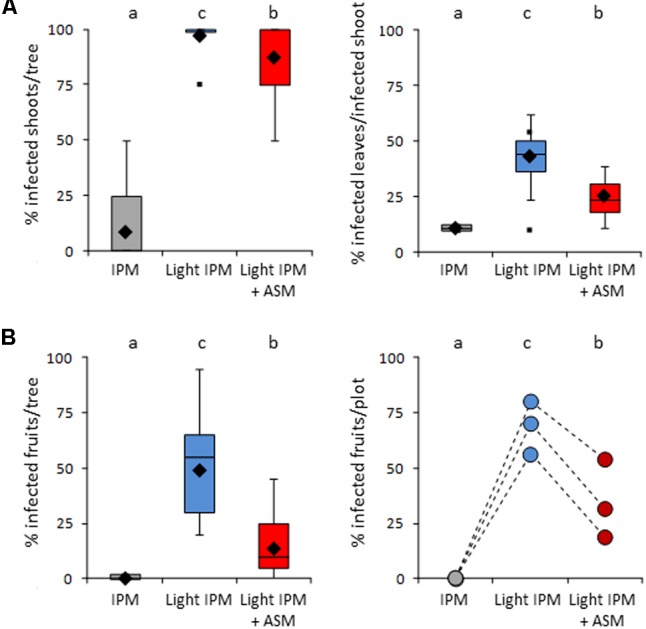
Protective effect of ASM against *Venturia inaequalis* in apple orchard. **(A)** Infection on shoots assessed in June: boxes represent values of percentage of infected shoots per tree (incidence, Left) and percentage of infected leaves per infected shoot and per tree (severity, Right). **(B)** Percentage of infected fruits assessed in June (Left) and after harvest (Right). In boxes, medians and means are indicated with horizontal lines and diamonds respectively. Boxes with the same letters represent medians that are not significantly different (*P* < 0.05, Kruskall–Wallis test, *n* = 33 (i.e., 11 trees × 3 plots) for **A** left, 9 ≥ *n* ≥ 33 for A right, *n* = 33 for **B** Left). At harvest, each point represents the percentage of infected fruits harvested from two trees per plot (**B** Right). ASM, acibenzolar-*S*-methyl; IPM, integrated pest management.

Disease contaminations of leaves (incidence and severity) and/or fruits (incidence) were assessed just after the end of the primary infection period (June) and at harvest (**Figure 2**). As expected, few apple scab symptoms were observed on leaves in the IPM strategy in which incidence and severity reached 8% and 11% respectively (**Figure 2A**). Conversely, strong disease development was recorded in the light IPM strategy with approximately 97% of incidence and 43% of severity. Thus, the disease pressure in the positive (IPM) and negative (light IPM) controls was appropriate to evaluate the efficacy of ASM treatments against apple scab. When combined to light IPM, ASM applications decreased slightly but significantly the incidence of the disease (around 87%) and had rather stronger effects on severity on leaves (around 25%) at the end of the primary contaminations. A strong decrease of incidence was observed on fruits at the end of the primary contaminations and after harvest (average incidence rates in light IPM + ASM: 13 and 34% respectively), in comparison to the light IPM (average incidence rates of 49 and 69% respectively; **Figure 2B**). However, the protection efficacy observed in the light IPM + ASM did not reach the protection obtained in the IPM control for which incidence and severity of apple scab did not exceed 10%, regardless of the organ (leaf or fruit) and the period of notation.

**FIGURE 3:**
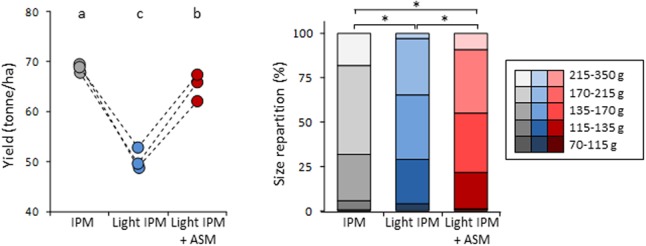
Effect of ASM on fruit yield **(Left)** and fruit caliber **(Right)**. **(Left)** Points represent values of yield per hectare and per plot. Values with the same letters are not significantly different (*P* < 0.05, Kruskall–Wallis test, *n* = 3). **(Right)** Star on two stacked bars indicates distributions significantly different (*P* = 0.05, χ^2^ test). ASM, acibenzolar-*S*-methyl; IPM, integrated pest management.

The yield of the orchard and the caliber of fruits were also compared between the three modalities (**Figure 3**). A slight but significant decrease of yield was observed in light IPM + ASM (65 ton/ha) in comparison to IPM control (69 ton/ha). In contrast a strong reduction was recorded in light IPM strategy (50 ton/ha). Differences were also noticed in the fruit calibers with the biggest fruits in IPM, the smallest in light IPM and an intermediate status in light IPM + ASM. Taken together, our results highlighted that the weak protection level provided by the light IPM affects the yield and the size of harvested fruits. Supplemented applications of ASM partially decreased the apple scab development, weakly affecting the yield and fruits size in that it diminished their caliber relative to the IPM control.

### Differential Responses of Apple Genotypes

Although encouraging, our field strategy requires further work for a reliable apple scab control that addresses economic concern in commercial orchards (mainly marketable healthy fruits). An ongoing question concerns the plant genotype effect, which is suspected to determine the plant’s ability to respond to PRI. We therefore investigated in semi-controlled conditions how the plant genotype affects the ability of ASM to elicit defense genes expression. Defense modulation by ASM was assessed in five apple genotypes (including Golden Delicious) displaying contrasting susceptibility to apple scab, and compared to water treated plants. Expressions of 28 defense genes were monitored before and at 3 dpt in the youngest expanded leaves of the plants, half of them having received an additional Hp treatment (see section “Materials and Methods”) at 1 dpt.

**FIGURE 4:**
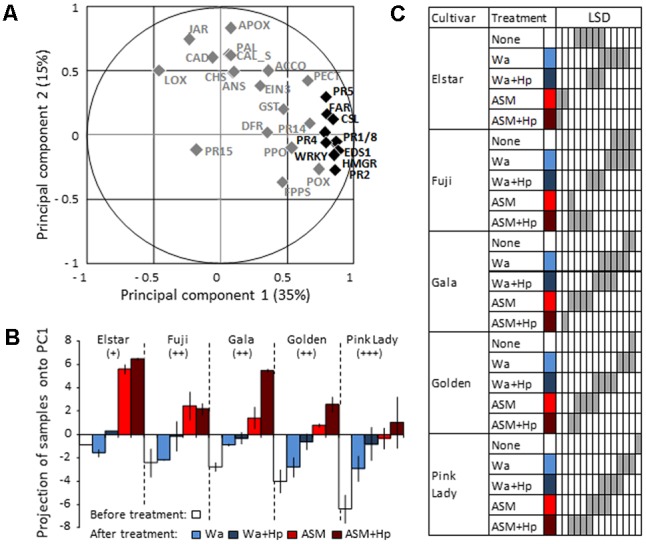
Principal component analysis (PCA) of defense gene expression in the five apple cultivars Elstar, Fuji, Gala, Golden Delicious (Golden), and Pink Lady before and at 3 dpt with ASM or water. At 1 dpt, half of each treated batch of plants received an additional hydrogen peroxide treatment to reveal priming effects. An untreated sample of Elstar was arbitrarily chosen as a unique calibrator for the calculation of the log_2_ ratio of each defense gene. **(A)** Variables (28 defense genes). **(B)** Projection of samples onto principal component 1 (PC1). Bars represent the mean of coordinates of two independent biological repeats, and extremities of vertical lines the two coordinates themselves. Apple scab susceptibility levels: +: moderately susceptible; ++: susceptible; +++: very susceptible. **(C)** Results of LSD test performed using data obtained with the 10 most important variables that explain PC1 (cos^2^ > 0.6), i.e., *PR1, PR2, PR4, PR5, PR8, HMGR, Far, CSL, EDS1, WRKY* (*n* = 20). Cultivars × Treatments sharing gray cells in the same sub column are not significantly different (*P* < 0.05). ASM, acibenzolar-*S*-methyl, Hp, hydrogen peroxide, Wa, water.

**FIGURE 5:**
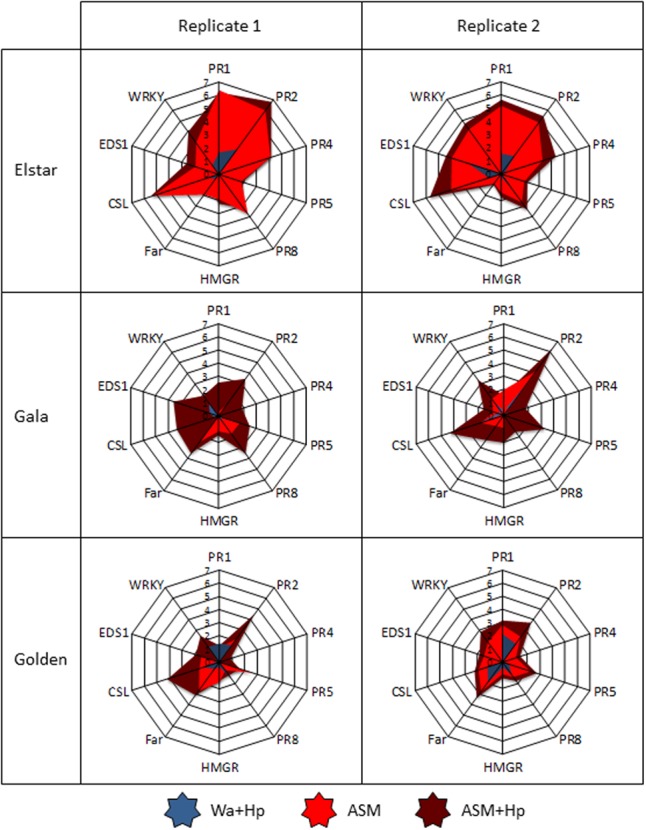
Focus on the log_2_ ratio of *PR1, PR2, PR4, PR5, PR8, HMGR, Far, CSL, EDS1, WRKY* genes in the three apple cultivars Elstar, Gala, and Golden in experiment 1 **(Left)** and 2 **(Right)**. Same data set than the one leading to **Figure 4** except that the log_2_ ratios were calculated relative to the water-treated leaves sampled at 3 dpt in each genotype and in each experiment. ASM, acibenzolar-*S*-methyl; Hp, hydrogen peroxide; Wa, water.

A PCA performed using genes as variables (**Figure 4A**) and samples as individuals (**Supplementary Figure S2**) revealed that the first component (35% of total variation) clearly discriminates treatments and correlates with *PR(1,2,4,5,8), CSL, HMGR, Far, WRKY*, and *EDS1* genes (*cos*^2^> 0.6). This allowed us to use principal component 1 (PC1) to summarize the data set. The comparison of PC1 coordinates of samples revealed different constitutive levels of defense (white bars in **Figure 4B**) among the five tested genotypes with three statistical groups (**Figure 4C**): the moderately susceptible Elstar variety with the highest steady-state level of defense, the susceptible Fuji/Gala/Golden varieties with intermediate levels, and the very susceptible Pink Lady variety with the lowest level. ASM significantly induced defenses in all genotypes in comparison to water treatment and this induction correlated with their initial level of defense (*r*^2^ = 0.90, *p* = 0.0133). Thus, the highest level of induction was observed in Elstar and the lowest in Pink Lady. Hp treatment on ASM treated plants revealed a significant priming effect of the PRI only in Gala and no correlation between final and initial levels of defense (*r*^2^ = 0.70, *p* = 0.0788). A focus on data obtained for Golden and for the two more reactive Gala and Elstar cultivars is presented as radar plots in **Figure 5**. It shows the differential amplitude of direct induction or priming of the ten selected genes in ASM treated tissues compared to water treated tissues in the two biological and independent replications. It highlights that amplitude of gene induction is much higher in Elstar than in Gala and Golden. This strong induction is highly reproducible and concerns all ten genes with the higher amplitude of induction recorded for *PR1* and *PR2*. When observed, amplification of defense responses due to an additional Hp treatment was very slight for the ten genes. By contrast, the pattern of gene induction for Gala and Golden was far more variable between the two replicates. This is particularly true for Gala where *PR1* and *PR2* are directly induced in one replicate while primed in the other.

### Persistent and Cumulative Effects

**FIGURE 6:**
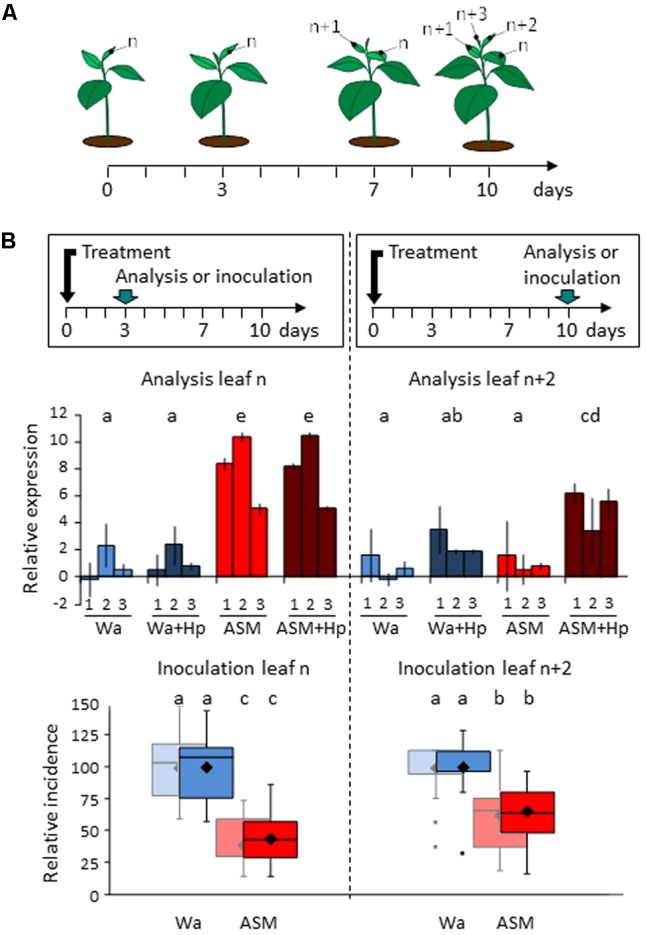
Persistence of action of ASM in apple seedlings. **(A)** Leaf numbering for experiments. **(B)** Analysis of relative expression of *PR2* (1), *PR4* (2), and *CSL* (3) (above) and protective effect against *Erwinia amylovora* (below). The youngest developed leaf of each seedling was either sampled or inoculated at 3 dpt (leaf n) or at 10 dpt (leaf *n*+2). For defense analysis, half of each block of plants received an additional hydrogen peroxide treatment 48 h before tissue sampling to reveal priming effects. Bars represent the mean of two independent biological repeats, and extremities of vertical lines the two values themselves. Log_2_ ratios were calculated relative to the mean of untreated leaves “*n*” at day 0 for each defense gene. For the three genes taken together, similar letters represent means that are not significantly different (*P* < 0.05, ANOVA and LSD test, *n* = 6). Boxes represent values of disease incidence assessed 2 (pastel colors) and 3 (bright colors) weeks after inoculation. Medians, means and outliers are indicated with horizontal lines, diamonds and squares respectively. Boxes with the same letters represent means that are not significantly different (*P* < 0.05, Kruskal–Wallis test, *n* = 9, i.e., 3 plots of 10 plants per experiment × 3 independent experiments). ASM, acibenzolar-*S*-methyl; Hp, hydrogen peroxide; Wa, water.

Our field strategy was an arbitrarily weekly-scheduled spraying of the PRI that encompassed the period of primary scab contamination and lead to a high number of applications (8). We wondered whether ASM is able to induce a persistent effect over a period of 6–8 days, especially in the young leaves newly developed after treatment. We therefore treated apple seedlings once with ASM or water and assessed the defense and resistance status of the youngest expanded leaf at 3 and 10 dpt (**Figure 6**). Following this procedure, leaves studied at 3 dpt received the treatments (leaf *n*), while 10 dpt-leaves did not (leaf *n*+2). We monitored their defense status by measuring the expression of three marker genes (*PR2, PR4*, and *CSL*) and assessed their resistance following a wound-inoculation with *E. amylovora.* Application of ASM considerably induced the defense levels in leaf n at 3 dpt, independently of Hp application performed 24 h before sampling. At 10 dpt, untreated leaves (*n*+2) behaved quite differently: ASM alone did not activate defense gene expression but clearly primed the defense responses as revealed by the Hp treatment performed 24 h before sampling. However, the final level of defense remained significantly lower in leaves *n*+2 than in leaves n. Resistance assessment to *E. amylovora* gave similar results: a single ASM treatment 3 days before inoculation provided a protection rate of roughly 60% vs. 35% when the treatment occurred 10 days before inoculation. These results confirm the ability of ASM to prime effective defenses in newly developed leaves and the difference in the protection rates recorded is consistent with the difference of amplitude of defense responses observed between the two leaf levels.

**FIGURE 7:**
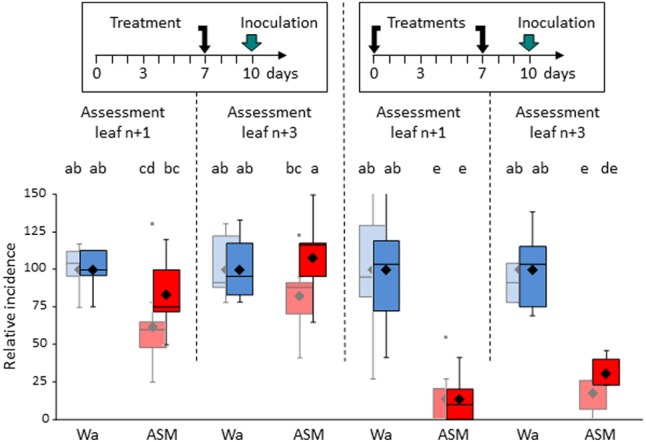
Efficacy of cumulative ASM treatments against *V. inaequalis* on apple seedlings. Same leaf numbering than in **Figure 6**. Boxes represent values of disease incidence assessed 2 (pastel colors) and 3 (bright colors) weeks after inoculation. Medians, means, and outliers are indicated with horizontal lines, diamonds, and squares respectively. Boxes with the same letters represent means that are not significantly different (*P* < 0.05, Kruskal–Wallis test, *n* = 9, i.e., 3 plots of 10 plants per experiment × 3 independent experiments). ASM, acibenzolar-*S*-methyl, Wa, water.

We next compared a single ASM application to two successive ASM applications (**Figure 7**). Seedlings were inoculated with *V. inaequalis* (spore-spraying on the whole plant) 3 days after the last treatment and sporulating lesions were recorded in two leaf levels corresponding to (i) the treated leaf (*n*+1) and (ii) the primed leaf (*n*+3).

A single ASM application allowed a protection rate of less than 50% in the treated leaf 2 weeks after inoculation, which decreases to around 20% and becomes no significant 3 weeks after inoculation. No significant protection was recorded in the primed leaf. By contrast, two successive ASM applications were far more effective than a single-one in terms of level and duration of protection, either in the leaf level primed by the first application and which received the second one (90% of protection at each disease assessment) or in the leaf level which did not received any product (around 75%). To confirm that plants are able to react to repeated applications of ASM, and especially top young leaves primed by previous ASM application, we inoculated similar leaves by *E. amylovora* (**Supplementary Figure S3**). Results showed a significant increase in resistance of ASM-primed leaves that were subsequently ASM-treated compared to ASM-primed leaves without further ASM application.

### Combination with Agricultural Inputs

**FIGURE 8:**
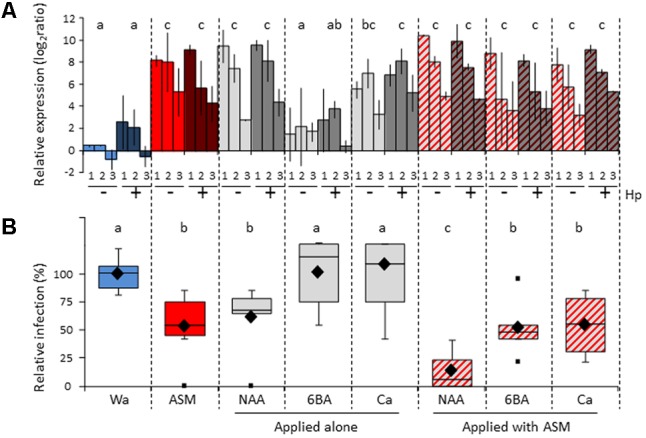
Effect of NAA, 6BA, and Ca applied alone or in combination with ASM on apple seedlings. **(A)** Relative expression of *PR2* (1), *PR4* (2), and *CSL* (3) in the youngest nearly expanded leaves at 3 dpt. At 1 dpt, half of each block of plants received an additional hydrogen peroxide treatment to reveal priming effects. Bars represent the mean of two independent biological repeats, and extremities of vertical lines the two values themselves. Log_2_ ratios were calculated relative to the mean of untreated youngest nearly expanded leaves at day 0 for each defense gene. Statistical analysis performed with values cumulated across the three genes (*n* = 6) and same letters represent means that are not significantly different (*P* < 0.05, ANOVA, LSD test). **(B)** Protective effect against *E. amylovora* inoculated into the youngest nearly expanded leaves at 3 dpt. Boxes represent values of disease incidence assessed 3 weeks after inoculation. Medians, means, and outliers are indicated with horizontal lines, diamonds and squares respectively. Boxes with the same letters represent medians that do not differ significantly (*P* < 0.05, Kruskal–Wallis test, *n* = 6, i.e., 3 plots of 10 plants per experiment × 2 independent experiments). ASM, acibenzolar-*S*-methyl, 6BA, 6-benzyladenine; Ca, calcium; Hp, hydrogen peroxide; NAA, naphthalene acetic acid; Wa, water.

In addition to pesticide, apple orchards are treated with diverse agricultural inputs like foliar fertilizers, growth regulators and thinning agents, all known to modify the physiological and hormonal state of the plant. Considering the numerous studies dealing with synergistic or antagonistic crosstalks between different phytohormone signaling pathways (Robert-Seilaniantz et al., 2011a), we wondered whether these specific agricultural inputs could have conflictive or additive interactions with PRIs, and whether these could affect the field efficiency of the latters. We choose three commercial products with a possible interfering role in plant defense: Rhodofix^®^, Maxcel^®^, and Pitpom^®^ whose active ingredients are respectively an auxin analog (Naphthalene acetic acid; NAA), a cytokinin analog (6-benzyladenine; 6BA) and calcium (Ca). In a first approach, we investigated on apple seedlings grown in semi-controlled conditions whether a combination of ASM with each of these products modifies its ability to induce defense or to protect against fire blight (**Figure 8**).

Relative expression of three defense genes (*PR2, PR4*, and *CSL*) was assessed at 3 dpt following single or combined applications of these compounds. When applied alone, the compounds ASM, NAA, and Ca significantly induced gene expression, whatever the gene considered, whereas 6BA had no effect on defense (**Figure 8A**). These results confirmed results obtained previously with NAA and 6BA (Dugé de Bernonville et al., 2014). When the three inputs were combined with ASM, levels of gene induction were significant and similar to those obtained with ASM alone. Hp application at 1 dpt revealed no priming effect, whatever the compound considered.

The fire blight protective assay was performed at 3 dpt on seedlings sprayed with compounds alone or combined with ASM. Fire blight relative infection was significantly reduced by ASM and NAA when applied alone (**Figure 8B**), again confirming previous results (Dugé de Bernonville et al., 2014). Combined application of ASM and NAA further enhanced the protection rate against fire blight, reaching 90% of efficiency. Neither 6BA nor Ca application resulted in significant disease control when sprayed alone and their association with ASM did not modify the protective properties of the latter. The additive effect between ASM and NAA was further confirmed in a single apple scab protective assay (**Supplementary Figure S4**). Taken together these results suggest that possible interactions may occur in orchard between ASM and other agricultural inputs.

## Discussion

The first objective of the work was to demonstrate that ASM, a PRI known for its strong performance in controlled conditions, could be part of apple scab management programs in the orchard. Our strategy allowed a significant control of the disease at the end of the primary contamination period, leading to a low incidence of fruit scab at harvest. It required eight successive applications of the PRI and avoided eight out of the twelve fungicides applied in IPM control plots during the same period. To the best of our knowledge, it is the first report of a protective effect of ASM against apple scab in the field, and more generally of the successful use of a PRI in an integrated apple scab program during the primary contamination period allowing a significant reduction of fungicides. Besides, such a spray schedule could be also useful to control fire blight, a disease difficult to manage due to its unpredictable and sporadic nature (Norelli et al., 2003). The sustained induction of resistance, especially during blossom period and on succulent young tissues growing afterward, should help to reduce host susceptibility to infection in case of fire blight outbreaks.

Acibenzolar-*S*-methyl eliciting ability was previously reported in different apple cultivars or seedlings such as Golden Delicious (Brisset et al., 2000; Hassan and Buchenauer, 2007), Gala (Aćimović et al., 2015), M26 rootstock (Baysal and Zeller, 2004; Abo-Elyousr et al., 2010) and Jonathan (Maxson-Stein et al., 2002). However, the comparison of several apple cultivars to ASM application has never been performed before. Our results suggest that the performance of ASM to control disease may be linked to a threshold of defense reached after ASM application in each genotype (taking into account their initial level of defense) rather to their ability in responding to the treatment. As an example, one can expect a much better performance of ASM in Elstar than in Pink Lady, in so far Pink Lady’s induced defense hardly reaches the level of constitutive defense of Elstar. It is also interesting to notice that the three intermediate genotypes (Fuji, Gala and Golden) belong to the same class of apple scab susceptibility as well as to the same class of constitutive defense. However, based on the enhanced ability of Gala to be primed by ASM, one can assume that our field strategy would have been even more efficient on this cultivar than on Golden Delicious. Such genotype comparisons should however be enlarged before giving recommendations on cultivars to favor or on the contrary to avoid when using PRIs in orchard management programs.

The priming effect of ASM has been reported several times, especially when used at low concentrations (50–100 μM) on parsley and Arabidopsis (Katz et al., 1998; Kohler et al., 2002). These authors observed that ASM has a dual role depending on which defense-related gene was considered. Some genes were directly induced (*POX, PR1*), while other genes were primed (*PAL*). Our work relies on a much higher concentration of ASM (near 1 mM) and a different subsequent stress (H_2_O_2_). In these experimental conditions, we also observed a dual role on defense-related genes but this was clearly linked to the genotype and to the ‘untreated *vs* treated’ state of the leaves rather than to the class of genes observed. The molecular bases of systemic priming by pathogens or PRIs have been shown to rely on the deposition of activating chromatin marks in the promoter region of defense genes, once SAR signal has been perceived. This is especially true for *WRKY* gene promoters in Arabidopsis and common bean, which accumulate various modified histones in remote leaves enabling sustained gene transcription upon subsequent stress perception (Jaskiewicz et al., 2011; Martínez-Aguilar et al., 2016). Whether accumulation of modified histone or other epigenetical changes (DNA methylation or nucleosome positioning, reviewed in Espinas et al., 2016) are involved in defense priming of perennial plants like apple still remains to be determined.

The priming of defense in top young leaves that emerged after ASM treatment is particularly interesting and underpins a biological reality since it is supported by our protection results, especially against *E. amylovora*. As aging tissues become physiologically resistant to many diseases (Develey-Rivière and Galiana, 2007) including apple scab and fire blight (Crosse et al., 1972; Jha et al., 2009), a systemic action of a PRI constitutes an interesting feature for the growers, sparing them to repeat treatments at each new leaf emergence. This is especially decisive in apple scab management with the primary scab period coinciding with the peak developmental time of shoots leading to a new leaf emergence every 3–4 days if optimal environmental conditions are present (Carissel et al., 2008). The present study suggests a probable decrease of defense responses after treatment with time and distance, i.e., in successive new leaves emerged after treatment. However, this decrease can be corrected by repeated applications of the PRI as highlighted in our experiments. In orchard, weekly scheduled applications of ASM seems therefore necessary at least at the beginning of the growing season and could perhaps become less frequent toward the end of the primary infection period as the rate of leaf emergence decreases.

Auxin is known for its antagonistic interaction with salicylate (SA)-signaling (Park et al., 2007; Wang et al., 2007; Robert-Seilaniantz et al., 2011b) while cytokinin and Ca^2+^ for their synergistic action with SA or ethylene (Raz and Fluhr, 1992; Choi et al., 2010; Liang et al., 2010; Li et al., 2016). In this study, we identified rather a strong additive action of the auxin analog NAA with the SA analog ASM in term of protection against disease and did not reveal any interference of the cytokinin analog 6BA or the Ca with the SA analog, although the later was able to induce apple defenses. This probably reveals that the genes selected in our study can be only considered as defense markers, but they are not involved in the resistance to *E. amylovora*. From a practical point of view, this work gives the first indications that agricultural inputs can interfere with ASM. This approach deserves to be further completed with (i) other inputs used in orchard (including pesticides, see for example Herms et al., 2002) and (ii) sequential applications (either before or after the PRI application) to ensure no conflicting interactions or to reveal useful additive ones. In this respect the treatment schedule performed during our field trial gives an example of how heavily trees are treated in conventional orchard management and demonstrates the need to address the question, especially on this crop.

Sustained activation of plant defense is often associated with a reduction of plant growth due to a growth-defense tradeoff which aims at prioritizing plant resource allocation (Huot et al., 2014 for a review). Repeated ASM treatments have for example been associated with wheat biomass reduction in the absence of disease or pest pressure (Heil et al., 2000). In our field trial, it is however impossible to assign the slight yield and caliber reduction observed in the light IPM + ASM strategy to a negative side-effect of ASM rather than to the residual disease impact. Only repeated ASM treatments combined with a full IPM strategy could help deciphering this concern.

Altogether, the present results provide important insights toward the way PRIs can be used in apple orchards in order to fight against threatening pests while reducing conventional pesticides use. Continuing to acquire knowledge on factors influencing PRI field efficiency will undoubtedly contribute to enhance their efficiency and thus popularize their use for safer agricultural practices. The influence of environmental conditions is notably an important question that remains to be addressed.

## Author Contributions

All authors listed have made a substantial, direct and intellectual contribution to the work, and approved it for publication.

## Conflict of Interest Statement

The authors declare that the research was conducted in the absence of any commercial or financial relationships that could be construed as a potential conflict of interest.

## Supplementary Material

FIGURE S1 Orchard plot layout. ASM, acibenzolar-*S*-methyl; IPM, integrated pest management.

FIGURE S2 Principal component analysis of defense gene expression in the five apple cultivars Elstar, Fuji, Gala, Golden Delicious (Golden), and Pink Lady before and 3 days after treatment with ASM or water. Half of each treated batch of plants received an additional hydrogen peroxide treatment 48 h before tissue sampling to reveal priming effects. Projection of samples onto principal components 1 and 2. An untreated sample of Elstar was arbitrarily chosen as a unique calibrator for the calculation of the 2^-ΔΔC_T_^ values of each defense gene. ASM, acibenzolar-*S*-methyl; Hp, hydrogen peroxide; Wa, water.

FIGURE S3 Efficacy of cumulative ASM treatments against *E. amylovora* on apple seedlings. Same leaf numbering than in **Figure 6**. Boxes represent values of disease incidence assessed 2 (pastel colors) and 3 (bright colors) weeks after inoculation. Medians, means and outliers are indicated with horizontal lines, diamonds and squares respectively. Boxes with the same letters represent means that are not significantly different (*P* < 0.05, Kruskal–Wallis test, *n* = 9, i.e., 3 plots of 10 plants per experiment × 3 independent experiments). ASM, acibenzolar-*S*-methyl, Wa, water.

FIGURE S4 Protective effect of NAA applied alone or in combination with ASM against *V. inaequalis* on apple seedlings, 3 weeks after inoculation. Each point represents the disease incidence recorded on a plot of 10 plants. Results from one experiment. The same letters represent means that are not significantly different (*P* < 0.05, Kruskal–Wallis test, *n* = 3, i.e., 3 plots of 10 plants in one experiment). ASM, acibenzolar-*S*-methyl; NAA, naphthalene acetic acid; Wa, water.

TABLE S1 Detailed treatment schedule performed over the season of primary contamination of apple scab in the experimental apple orchard in IPM, light IPM and light IPM + ASM strategies. ASM, acibenzolar-*S*-methyl; IPM, integrated pest management.
