# Supplementary figures and images for: When a Plant Resistance Inducer Leaves the Lab for the Field: Integrating ASM into Routine Apple Protection Practices
Source: Front Plant Sci. 2017 Dec 4;8:1938. doi: 10.3389/fpls.2017.01938 (PMC5723009; doi:10.3389/fpls.2017.01938)

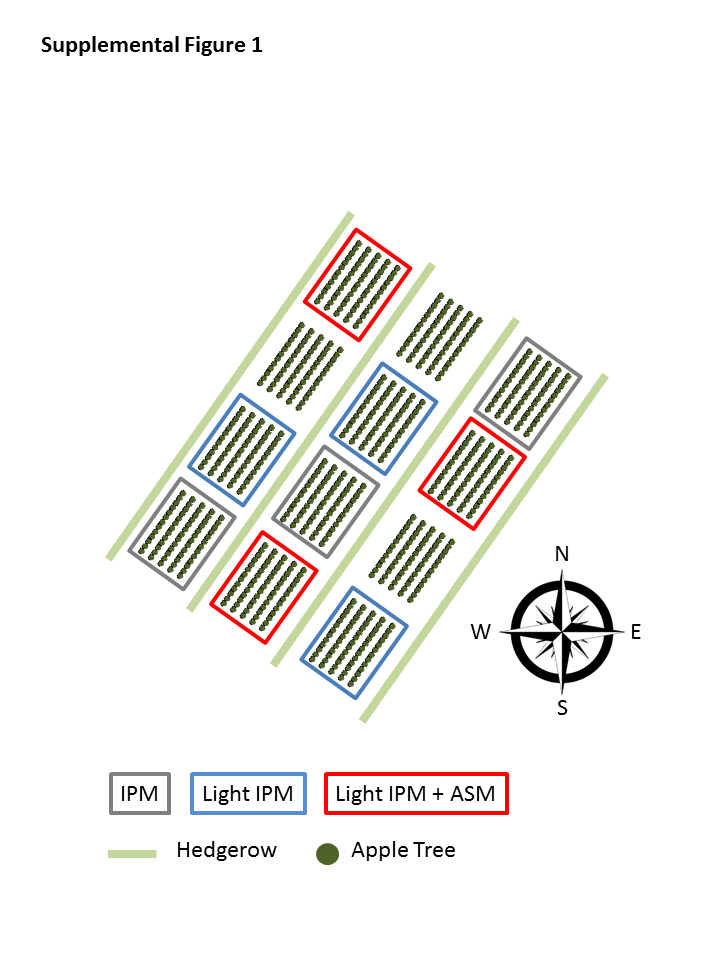

Supplement: FIGURE S1 — Orchard plot layout. ASM, acibenzolar-S-methyl; IPM, integrated pest management. [file Image_1.TIF]

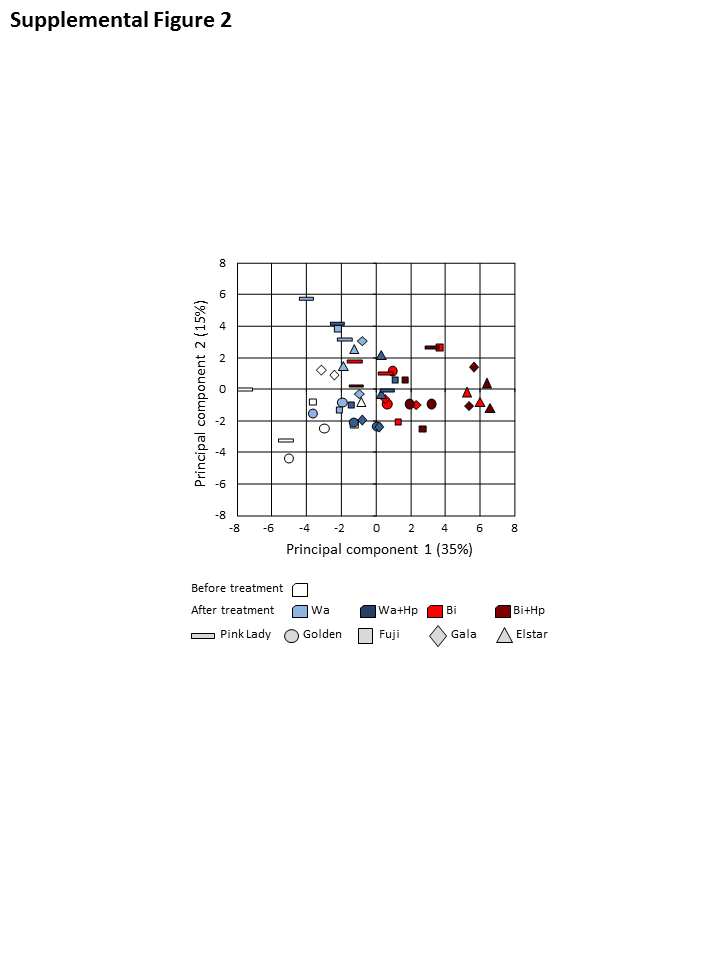

Supplement: FIGURE S2 — Principal component analysis of defense gene expression in the five apple cultivars Elstar, Fuji, Gala, Golden Delicious (Golden), and Pink Lady before and 3 days after treatment with ASM or water. Half of each treated batch of plants received an additional hydrogen peroxide treatment 48 h before tissue sampling to reveal priming effects. Projection of samples onto principal components 1 and 2. An untreated sample of Elstar was arbitrarily chosen as a unique calibrator for the calculation of the 2-ΔΔCT values of each defense gene. ASM, acibenzolar-S-methyl; Hp, hydrogen peroxide; Wa, water. [file Image_2.tif]

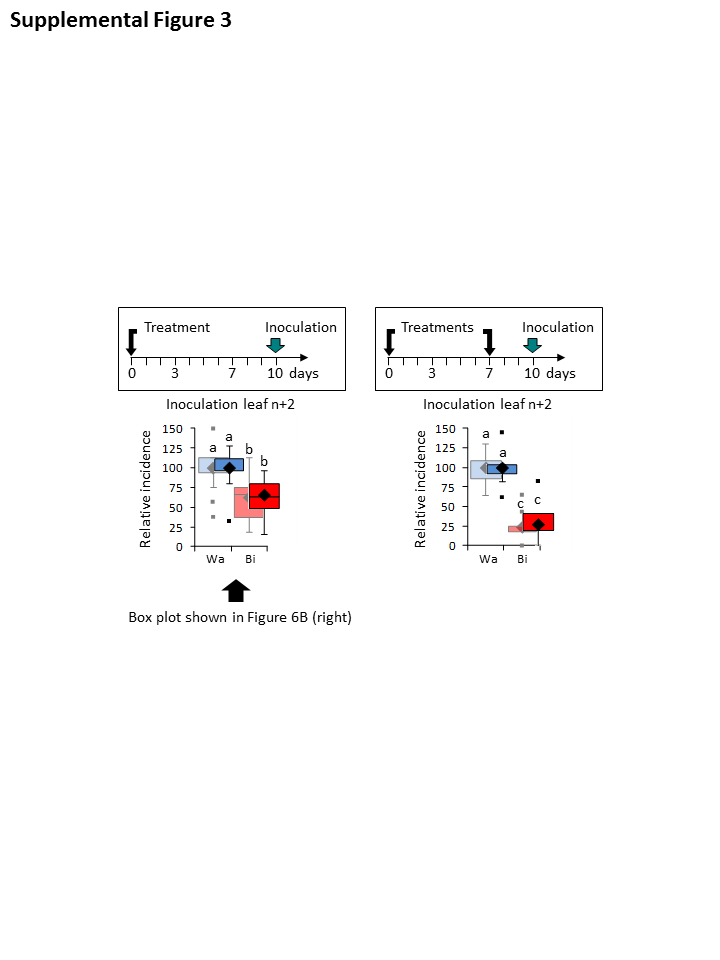

Supplement: FIGURE S3 — Efficacy of cumulative ASM treatments against E. amylovora on apple seedlings. Same leaf numbering than in Figure 6. Boxes represent values of disease incidence assessed 2 (pastel colors) and 3 (bright colors) weeks after inoculation. Medians, means and outliers are indicated with horizontal lines, diamonds and squares respectively. Boxes with the same letters represent means that are not significantly different (P < 0.05, Kruskal–Wallis test, n = 9, i.e., 3 plots of 10 plants per experiment × 3 independent experiments). ASM, acibenzolar-S-methyl, Wa, water. [file Image_3.tif]

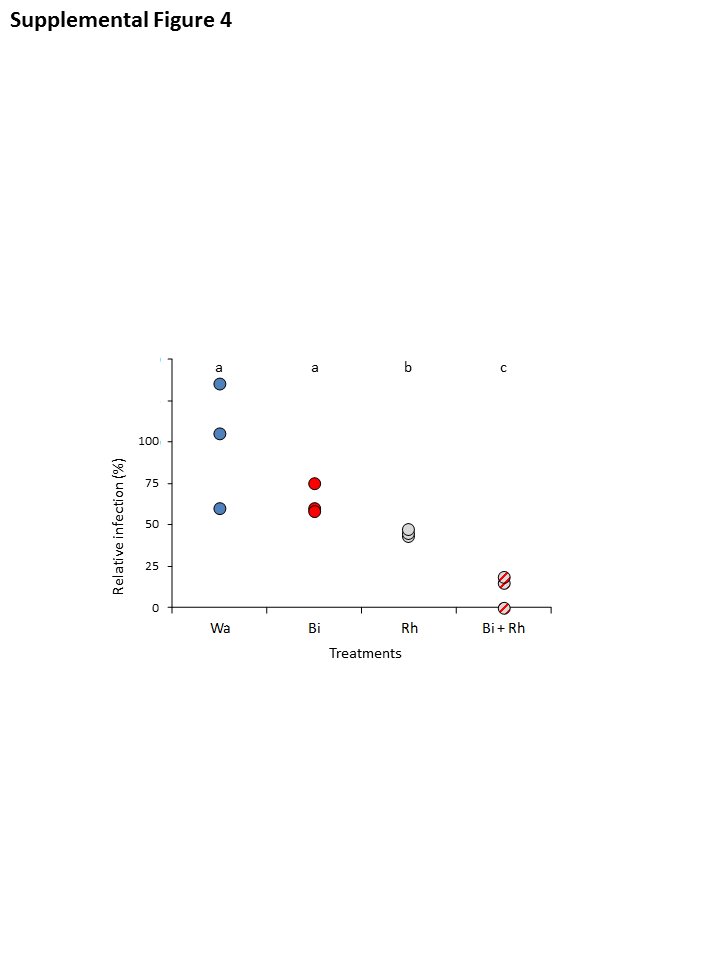

Supplement: FIGURE S4 — Protective effect of NAA applied alone or in combination with ASM against V. inaequalis on apple seedlings, 3 weeks after inoculation. Each point represents the disease incidence recorded on a plot of 10 plants. Results from one experiment. The same letters represent means that are not significantly different (P < 0.05, Kruskal–Wallis test, n = 3, i.e., 3 plots of 10 plants in one experiment). ASM, acibenzolar-S-methyl; NAA, naphthalene acetic acid; Wa, water. [file Image_4.tif]
